# Unraveling the Place of Small Molecules in the Treatment of Fistulizing Crohn’s Disease—A Systematic Review and Network Meta-Analysis

**DOI:** 10.1093/crocol/otad074

**Published:** 2023-12-13

**Authors:** Mohamed Attauabi, Ditlev Nytoft Rasmussen, Fredrik Olof Bergenheim, Johan Burisch, Jakob Benedict Seidelin

**Affiliations:** Department of Gastroenterology and Hepatology, Copenhagen University Hospital—Herlev and Gentofte, Herlev, Denmark; Gastrounit, Medical Section, Copenhagen University Hospital—Amager and Hvidovre, Hvidovre, Denmark; Copenhagen Center for Inflammatory Bowel Disease in Children, Adolescents, and Adults, Hvidovre Hospital, Hvidovre, Denmark; Department of Gastroenterology and Hepatology, Copenhagen University Hospital—Herlev and Gentofte, Herlev, Denmark; Department of Gastroenterology and Hepatology, Copenhagen University Hospital—Herlev and Gentofte, Herlev, Denmark; Gastrounit, Medical Section, Copenhagen University Hospital—Amager and Hvidovre, Hvidovre, Denmark; Copenhagen Center for Inflammatory Bowel Disease in Children, Adolescents, and Adults, Hvidovre Hospital, Hvidovre, Denmark; Department of Gastroenterology and Hepatology, Copenhagen University Hospital—Herlev and Gentofte, Herlev, Denmark

With interest, we read the review by Dr. Singh et al. outlining the available medical options for perianal fistulizing Crohn’s disease (CD).^1^ We would like to clarify the comparative efficacy and ranking of available interventions, with an emphasis on small molecules, which was not analyzed in the review.

We conducted a Bayesian random-effects network meta-analysis (NMA) to account for trial heterogeneity, handling varying follow-ups in each trial as recommended.^2^ As of July 10, 2023, we identified 21 randomized controlled trials comprising 1452 patients. Compared to another NMA,^3^ we included 4 studies of stem cells,^4–7^ and 1 study each of upadacitinib,^8^ ustekinumab,^9^ and adalimumab versus ustekinumab.^10^ Based on CINeMA, confidence in findings was rated high between the active agents and placebo but low between active agents.

Regarding induction of fistula remission during Weeks 6–14, defined as closure of all external openings draining at baseline, infliximab 5 mg/kg with ciprofloxacin (surface under the cumulative ranking [SUCRA] 0.895, hazard ratio [HR] = 14.80 [95% credible intervals] 2.83–88.92) or monotherapy (SUCRA 0.670, HR = 5.76 [1.88–21.62]) and adipose-derived stem cells (ASCs)-Cx401 (SUCRA 0.757, HR = 9.41 [1.28–173.76]), ranked highest and superior to placebo. Upadacitinib 45 mg also exceeded placebo significantly (SUCRA 0.516, HR = 3.69 [1.09–17.47]), while ustekinumab 6 mg/kg (SUCRA 0.452, HR = 2.33 [0.91–6.20]) and vedolizumab 300 mg (SUCRA 0.339, HR = 1.05 [0.72–20.50]) did not.

Regarding maintaining fistula remission through Weeks 44–56, upadacitinib 30 mg (SUCRA 0.750) or 15 mg (SUCRA 0.721) ranked highest. Despite significant efficacy in the trial (30 mg: 4/19 (21.1) versus 0/25, *P* = .029; 15 mg: 6/35 (17.1), *P* = .036),^8^ upadacitinib did not outperform placebo in the NMA (Figure 1). Adalimumab 80/40 mg (SUCRA 0.542), infliximab 5 mg/kg (SUCRA 0.471), and ASCs-Cx601 (SUCRA 0.403) were superior to placebo (Figure 1), while vedolizumab 300 mg and ustekinumab 6 mg/kg were not. None of the interventions separated significantly from the others in both NMAs. No data on the benefit of adding ciprofloxacin to TNF antagonists for this NMA were retrieved. Limitations include the inclusion of anti-TNFs refractory patients in the study of ASCs-Cx601,^4^ and heterogeneity in terms of dedicated or post hoc trial analyses.

**Figure 1.**
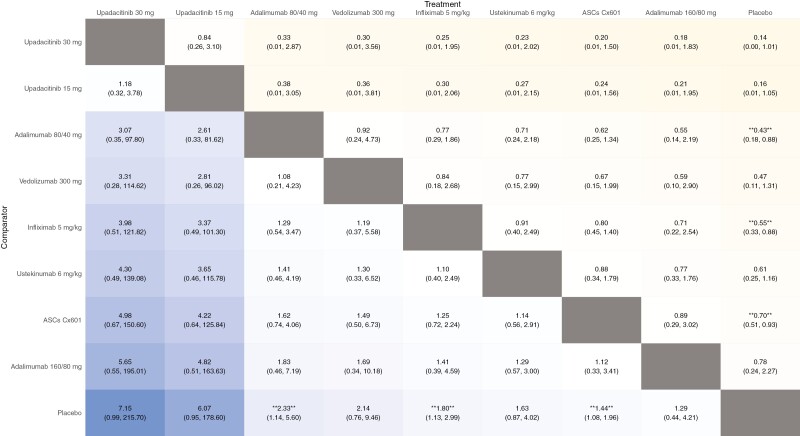
League plot for the comparison of advanced therapies in patients with fistulizing Crohn’s disease for maintenance of fistula remission at Weeks 44–56. The values in each cell represent the relative treatment effect (hazard ratio and 95% credible intervals) of the column-defining treatment compared to the row-defining treatment. A double asterisk indicates statistical significance. ASCs, adipose-derived stem cells.

In conclusion, infliximab 5 mg/kg, ASCs, or upadacitinib 45 mg may be the best option for achieving fistula remission in patients with CD, despite no significant differentiation from other agents.

Dr. Singh et al. are commended for their important endeavor. Nevertheless, the absence of data on upadacitinib or comparative analyses undermines the ability to provide guidance for well-informed treatment choices.

## Data Availability

Data are presented in the current manuscript, its Supplemental Digital Content, or within the manuscripts or appendices of the included studies.
